# Biomimetic Nanovesicles—Sources, Design, Production Methods, and Applications

**DOI:** 10.3390/pharmaceutics14102008

**Published:** 2022-09-22

**Authors:** Marcel Franco Mougenot, Vanessa Sousa Pereira, Ana Letícia Rodrigues Costa, Marcelo Lancellotti, Marimelia Aparecida Porcionatto, Juliano Coelho da Silveira, Lucimara Gaziola de la Torre

**Affiliations:** 1 Department of Materials and Bioprocesses Engineering, School of Chemical Engineering, University of Campinas, Campinas 13083-970, Brazil; 2 Institute of Exact and Technological Sciences, Campus Florestal, Federal University of Viçosa (UFV), Florestal 35690-000, Brazil; 3 Faculty of Pharmaceutical Sciences, University of Campinas (UNICAMP), Campinas 13083-871, Brazil; 4 Department of Biochemistry, Escola Paulista de Medicina, Universidade Federal de São Paulo, São Paulo 04039-032, Brazil; 5 Department of Veterinary Medicine, Faculty of Animal Sciences and Food Engineering, University of São Paulo, Pirassununga 13635-900, Brazil

**Keywords:** liposome, drug delivery, gene delivery, vaccine, nanotechnology, extracellular vesicles

## Abstract

Despite all the progress in the field of liposomes and nanoparticles for applications as drug and gene delivery systems, the specific targeting and immune system escape capabilities of these systems are still limited. Biomimetic nanovesicles emerged as a strategy to overcome these and other limitations associated with synthetic carriers, such as short circulation time, cytotoxicity, and difficulty in crossing biological barriers, since many of the desirable abilities of drug delivery systems are innate characteristics of biological vesicles. Thus, the question arises: would biomimetic nanovesicles be responsible for addressing these advances? It is currently known that biomimetic nanovesicles (BNV) can combine the intrinsic advantages of natural materials with the well-known production methods and controllability of synthetic systems. Besides, the development of the biotechnology and nanotechnology fields has provided a better understanding of the functionalities of biological vesicles and the means for the design and production of biomimetic nanovesicles (BNV). Based on this, this work will focus on tracking the main research on biomimetic nanovesicles (BNV) applied as drug and gene delivery systems, and for vaccines applications. In addition, it will describe the different sources of natural vesicles, the technical perspectives on obtaining them, and the possibility of their hybridization with synthetic liposomes.

## 1. Introduction

Biomimetic nanovesicles (BNV) are a multidisciplinary field involving engineering, medicine, chemistry, and biology. BNV can be defined as nanomaterials synthesized by artificial, biological, or combined routes mimicking natural ones. Thus, studying these nanostructures involves their design, synthesis, characterization, biological function, and application. This approach combines the intrinsic advantages of natural materials like specific targeting and biocompatibility, with the well-known production protocols and high drug load capability of synthetic materials.

**Figure 1:**
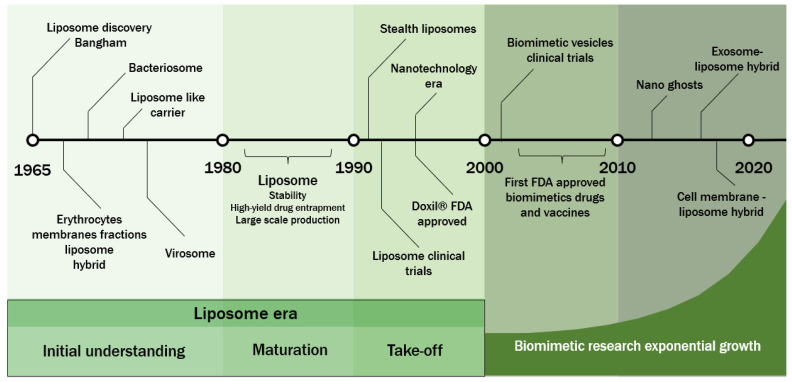
Timeline of the achievements in liposomes and biomimetic vesicles, including the main milestones from the discovery of liposomes by Bangham in 1965, passing through the phases of the liposome era, and the last two decades of exponential growth in biomimetic nanovesicles research.

Advances in biomimetic vesicles require a deep knowledge of liposomes. A timeline of the main achievements in this until now are presented in Figure 1. These first nanovesicles were discovered by Alec D. Bangham and dated 1965 [1,2]. At this date, the reason for the study was to create a simulacrum of a cell membrane to understand the mass transfer phenomena through it [2,3]. Liposomes are formed by mixing amphiphilic phospholipids with an excess of aqueous media. In this medium, the lipids spontaneously self-organize into an approximately spherical shape to form a lipid bilayer membrane and an aqueous core. This self-aggregate system occurs due to a more thermodynamic favorable state when the attractive forces between the hydrophobic lipid tales lead them to less exposure to water molecules, and the aqueous phase stabilizes the hydrophilic lipid headgroup [3,4].

For almost 60 years, the liposome has been the most researched nanovesicle and approved drug delivery system for clinical use [5]. In this review, the term “synthetic membrane” will describe liposomes obtained from conventional techniques using purified lipids to differentiate from natural membranes obtained from living organisms. Besides the recent advances in biomimetic vesicles, from the beginning of liposome discovery, scientists tried to imitate and use nature to functionalize nanoparticles (NP) with specific characteristics. In 1969, antigens from methanol-soluble fractions of sheep erythrocytes membranes were incorporated into liposomes and could bind with antibodies [6]. In 1971, the first hybrid between liposome and bacteria was synthesized with Lipid A and lipopolysaccharides from *Salmonella minnesota*, giving them immune sensitivity when exposed to serum with specific antibodies [7,8]. In 1975, for the first time, biomimetic nanovesicles were produced using transmembrane proteins from a virus and conventional lipids. The nanostructures were called virosomes by the authors. Hemagglutinin and neuraminidase from the influenza virus were allocated in the membrane of unilamellar liposomes. The biospecificity of virosomes was proved since they exhibited aggregation when treated with influenza-A antiserum [9].

The 1980s marked the maturation of liposome technologies, with important advances in stability, high-yield drug entrapment, and large-scale production processes. In the 1990s, liposomes took off with the initial clinical trials and the first FDA-approved liposome drug (Doxil^®^). During this period, the discovery of and progress in stealth liposomes also occurred [1,10,11].

The 2000s arrived with the beginning of clinical trials using biomimetic-inspired drugs for vaccines, drugs, and gene delivery. During this decade, a series of biomimetic virus-inspired medicines were approved by the FDA (Gardasil^®^, Cervarix^®^, Epaxal^®^, Invivac^®^, and Inflexal V^®^). Among its achievements, these new drugs proved the concept of the two virus-inspired vesicle approaches i.e., virus-like particles (VLP) and virosomes. Over the past two decades, the interest in biomimetic drugs has grown exponentially. The research’s efforts can be seen in all sources of vesicles, like viruses, bacteria, and animal cells [12,13,14,15,16,17].

Whatever the progress in the field of liposomes and nanoparticles for drug delivery systems, the technology is far from achieving Paul Ehrlich’s dream—the magic bullet. Especially in specific targeting and escape from the immune system capabilities, the drug delivery systems have much work ahead. Could biomimetic nanovesicles be responsible for addressing these advances? To address this important question this review will focus on presenting the different sources of natural vesicles, like mammalian cells, bacteria, and viruses. In addition, technical perspectives in obtaining these materials and the possibility of hybridization with conventional liposomes will also be discussed. This review article will not focus on another strategy to achieve specific targeting involving chemical modification of lipids, attachment of antibodies, and ligand proteins conferring properties in the conventional vesicles. The reader can find an interesting review about lipid chemical modification in Almeida et. al, Lima et al. and Cao et al. work [5,18,19].

## 2. Natural Membrane Sources

**Figure 2:**
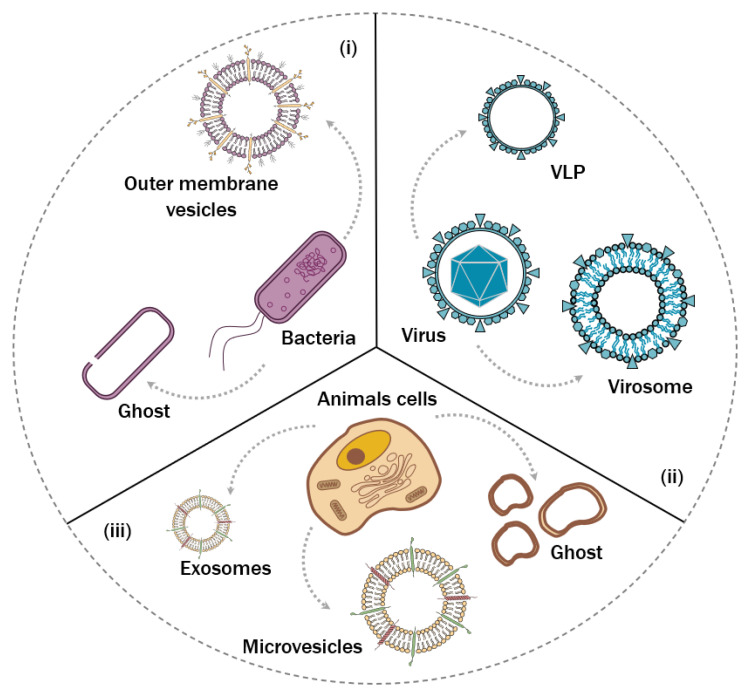
Natural membrane sources and their origin. (**i**) Outer membrane vesicles are nanovesicles naturally secreted by bacterial culture and ghosts are obtained by removing the internal contents of bacteria. (**ii**) Viruses are employed to produce virus-like particles (VLPs) or virosomes, the latter by combining their surface protein with commercial lipids. (**iii**) Mammalian cells are used to produce exosomes and microvesicles, naturally secreted by cell physiology, as well as to obtain cell ghosts by removing their internal content.

Natural vesicles or membranes have biospecific molecules to promote cell-to-cell communication and invasion into mammalian cells or bacteria. Inspired by these natural events, an exciting strategy is to use the whole natural membrane or a purified protein from the natural source instead of obtaining a chemical modification of a conventional lipid. Viruses, bacteria, mammalian cells, and extracellular vesicles from different types of cells can be used as a source of natural material to be used as a drug delivery system or combined with synthetic membranes (Figure 2). Yeast is another potential alternative, but the use of this source as a drug delivery system lies in the early stages compared to those. More information about yeast for drug delivery application can be found in Tan et al. work [20].

### 2.1. Virus

In the last decades, different viruses have emerged and played a key role in public health—challenging the human species to undertake a rapid response. Human immunodeficiency virus (HIV), influenza virus, Ebola virus, Zika virus, and most recent coronavirus COVID-19 required a tremendous effort to develop vaccines, medicines, infrastructures, and strategies to combat these diseases [21,22].

The virus’s morphology broadly consists of an external structure composed of proteins, named capsids, that enclose the genetic material (RNA) in the core. Due to simplistic assembly, viruses do not have arrangements to self-replicate. Therefore, they must invade a cell and use the host’s machinery to replicate [21]. To accomplish this task in more complex organisms, some viruses develop surface proteins that help them to evade the immune system. Several viruses can also target specific organs or cells [23,24,25]. All these capabilities, site-specific targeting, escaping the immune system, and entering the cell, make viruses a promising candidate for developing new biomimetic nanovesicles for drug delivery systems.

The two major groups of BNV inspired by viruses are virus-like particles (VLP) and virosomes [26]. A virus-like particle (VLP) is a self-assembled structure formed by capsid proteins that mimic the mother virus. A virosome is a liposome synthesized with virus proteins into the phospholipid bilayer. Both consist of spherical nanostructures with a hollow core and without virus genetic material (RNA). These vesicles can carry drugs, including chemotherapeutics such as doxorubicin and monomethyl auristatin, antibodies, nucleic acids, proteins, peptides, carbohydrates, contrast agents, and even coat other nanoparticles [24,25,26,27].

There are two routes to producing virus functional components: biotechnological and synthetic. The biotechnological route is the most used and common method and comprises a cell culture (e.g., yeast, mammalian cells, chicken egg, and others), inoculated by the virus of interest. Then the medium is collected, purified, and washed with an appropriate detergent to remove the envelope components (soluble) from the genetic material (insoluble). After the detergent removal, the functional components can be used. The synthetic approach synthesizes artificial molecules that mimic the virus capsid [25,26,27].

### 2.2. Outer Membrane Vesicles (OMV)

OMVs are spherical nanoparticles with sizes between 30–200 nm, produced by all types of Gram-negative bacteria [28,29,30]. Although discovered in 1967 [31], it was only in the last two decades that these biological vesicles have become more researched. Recently, OMVs have been used in pharmaceutical applications such as vaccines, drugs, and gene delivery [12,28,32,33].

The OMVs’ biogenesis occurs when the bacterial outer membrane detaches from the peptidoglycan (PPG) layer [34,35]. After an outward budding from the bacterial outer membrane, the OMVs are released into the extracellular medium [32]. Thus, as in its origins, the OMVs membrane is constituted of lipids and proteins [33].

The principal lipids in the membrane of OMVs are phospholipid and lipopolysaccharide (LPS). The phospholipids most commonly present in the membrane are phosphatidyl ethanolamine and phosphatidyl glycerol, varying according to the species [33]. LPS, also called endotoxin, is well known for the pro-inflammatory stimulus for the immune system in mammals. However, at low doses, a certain amount of LPS can help the immune system by stimulating polyclonal differentiation, multiplication of B-cells to secrete antibodies, and even playing an essential role in developing healthy immune systems in children [36].

A wide variety of proteins are present in the membrane or OMVs. Porins are one of the most abundant proteins in the membrane of OMVs. These proteins may involve specific adherent-invasive functions in host cells and immunostimulatory activities. Other proteins responsible for the adherent-invasive capability in OMVs are the adhesins, like Ail, IpaB, IpaC, and IpaD. Also present in OMVs: murein hydrolases and endopeptidase involved in competing mechanisms with other bacteria species, multi-drug efflux proteins responsible for antibiotic resistance, lipoproteins that can cause inflammatory responses, and virulence factors like toxins and digestive enzymes [12,37].

Bacterial culture is the only way to obtain OMVs, and the production is part of Gram-negative bacteria’s normal physiology. However, many events can trigger and increase OMVs production, such as stress during culture, high or low temperature, nutrient deficiency, and the presence of antibiotics [28,29,34]. After culturing, it is necessary to separate the OMVs from the bacterial culture, and several methods can be applied, such as ultracentrifugation, ultrafiltration, and protein precipitation. If necessary, detergent can be used in the separation step to remove or attenuate the LPS presence [28,32,33].

### 2.3. Bacterial Membrane (Bacterial Ghosts or Bacteriosomes)

Bacteria are well known for their immunostimulation qualities, but recently bacterial membranes (or bacterial ghosts, or bacteriosomes) have been explored as vesicles for drug and gene delivery applications. The basic structure of a bacterial ghost (BG) is a membrane bacteria envelope with channels (or pores) formed by chemical or genetic engineering methods. These vesicles can be loaded with chemical drugs such as doxorubicin or DNA to treat tumors, inflammation, infection, and vaccines [38,39,40].

It is fundamental for BGs production to create pores in the bacteria membrane capable of eliminating the internal content (nucleic acids, ribosomes, metabolites, and other components). BGs may be produced by two approaches: chemical or genetic engineering routes.

The chemical methods use some substances, such as model amphiphilic peptide (MAP) dissolved in Na_2_HPO_4_, or chemicals, such as NaOH, SDS, and H_2_O_2_, to create channels between the bacterial membrane. Although it is simple, rapid, and does not change the bacteria’s three-dimensional morphology, the chemicals can sometimes damage the wall and create malfunctioning or weak points. An advantage of this method is the possibility to apply it to Gram-negative and Gram-positive bacteria [38,39].

Another approach involves genetic engineering by controlling the expression of the cloned lysis gene E. The expression of the gene E leads to the biosynthesis of the protein E, which oligomerizes and fuses the inner and outer membranes, thus creating transmembrane tunnels. Therefore, the cytoplasmic contents are released, and the bacterial membrane remains intact. This method is only applied to Gram-negative bacteria since the genetic modification of gene E leads to Gram-positive bacteria death. The genetic engineering method is the most used route for producing BGs, and part of this can be justified due to a more unchanged membrane [38,39,40].

A bacterial ghost has the same basic structure and composition as the mother cell, except for the tunnels created to release the internal content. The size of the BGs varies between 0.5–2 µm, and the tunnel formed by E-lysis ranges between 40 and 200 nm and does not show a stable structure [39,40,41]. Also, extrusion can be used to create nanovesicles to reduce the size of BGs [42]. The ghost membrane is composed of phospholipids, lipopolysaccharides (LPS), membrane proteins, lipoproteins, monophosphoryl lipid A, peptidoglycan (PPG), or flagella [36,37,38]. Depending on the applications, OMVs are more suitable than BGs since OMVs do not have the peptidoglycan (PPG) layer [39,40,41].

### 2.4. Extracellular Vesicles (EV) from Mammalian Cells

Extracellular vesicles (EV) are membrane-enclosed vesicles secreted by all cell types during their normal physiology or abnormalities. EVs are generally divided into three groups according to their size and biogenesis: exosomes are generated by endosomal pathway (50 to 150 nm), microvesicles are formed from direct outward budding of the plasma membrane (50 to 1000 nm), and apoptotic bodies originate during the cell death (50–5000 nm). Since only size is not enough to classify the type of vesicles, it is recommended to call extracellular vesicles followed by the detailed isolation protocol (MiSEV2018). The overlap between exosome size and microvesicle size scale makes it difficult to separate and purify these extracellular vesicles. Many authors use exosomes to describe a mixture of exosomes and microvesicles with a size <200 nm. However, to avoid misunderstanding the appropriate description of this mixture is small extracellular vesicles (sEVs). To better characterize extracellular vesicles, it is imperative to consult “Minimal information for studies of extracellular vesicles 2018” (MISEV2018) [43].

Especially exosomes and microvesicles have drawn research attention to drug and gene delivery applications [44,45,46].

Exosome biogenesis initiates endocytosis when the plasma cell membrane invaginates, forming the early sorting endosomes (ESEs). During this process, extracellular content is encapsulated in the lumen of the ESEs, and the plasma membrane constitutes its boundary. ESEs mature and lead to late sorting endosomes (LSE). Eventually, a new invagination in the LSE generates the multivesicular bodies (MVB), containing intraluminal vesicles (ILVs). If the MVBs fuse with the plasma cell membrane, the ILVs are released into the extracellular medium; they are now called exosomes [44,45,46]. Due to the endocytosis pathway, the exosome membrane is distinct from the plasma cell membrane.

The membrane composition and cargo of extracellular vesicles are highly heterogeneous, and their functionality varies according to the cell of origin (source) and the recipient cells. The EVs membrane is a lipid bilayer with surface proteins such as tetraspanins, integrins, immunomodulatory proteins, and others [45].

The major challenge in addressing extracellular vesicle technology is the low throughput of production and isolation protocols [46,47]. To produce EVs it is necessary to cultivate cells and collect the medium where the EVs are secreted. The EVs are isolated from the medium by ultracentrifugation and ultrafiltration with tangential flow filtration, among others. In the last few years, the effort to increase the EVs production can be divided into two groups: the first scales up the production step (cell culture), and the second works to increase the EVs production yield per cell [47].

### 2.5. Mammalian Cell Membranes

An alternative to conventional drug delivery systems is the cell membrane as a structural component for developing new particles capable of incorporating active compounds or genetic material [48,49]. Conversely, particular membrane proteins can also be used as a component of conventional nanoparticles [50]. This delivery system may mimic cellular structural aspects and deliver drugs to target locations. Then, using plasma membrane or specific membrane proteins of different mammalian cells emerges as an alternative to using EVs and exosomes.

Membrane compounds can be obtained using cell lysis methods and then, in an additional step, reducing its size and stabilizing it with chemical modifications, such as polyethylene glycol (PEG). There are available protocols for cell processing, removing intracellular contents, and preserving the membranes [48,49,51]. In this sense, a common term used is “ghost cell”.

The term “ghost cell” was firstly introduced by Shattuck (1928) in a comparative study on the hemolysis of chicken blood red blood (nucleated and ovals) and mammalian blood (anucleated and flattened) [52]. The term “ghost cell” was also redefined by Hoffman (1958) for red cells (red blood cells or erythrocytes) that lost their hemoglobin. In addition, a “ghost cell” also means a dead cell that remains visible without presenting evidence of nucleus and cytoplasmic content. Hoffman (1958) also defined the “ghost system” for human red blood cells as being without cytoplasm. Although the ghost cells have properties of plasma membranes of intact cells, the author states that there is dependence on the method to obtain them, which motivated him to investigate red blood cells through hypotonic hemolysis [53].

The classical method for obtaining ghost cells from red blood cells is cell rupture in a hypotonic solution, and homogenization followed by centrifugation steps, promoting the elimination of hemoglobin and cytoplasmic content. The advantage of this type of cell is the absence of a nucleus, making the method less complicated. Although the application of ghost cells has not been for drug delivery, the protocol to obtain them has already begun to be developed, paving the way for future applications in drug delivery.

In this context, a new class of drug carriers derived from the classic ghost cells of platelets [50], leukocytes [54], mesenchymal stem cells [49,55], and cancer cells [56] have also used membrane components for the delivery of drugs, suggesting the potentiality of using different cells as a source of information for drug delivery. Research in this field also reveals that membrane constitution and properties can be modulated depending on how cells are cultured or even stressed, leading to different biological performances [57].

## 3. Design Strategies for Biomimetic Vesicles

**Figure 3:**
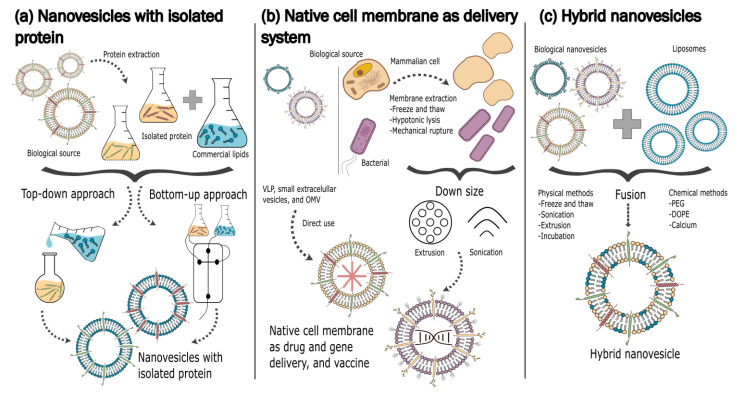
Design strategies for biomimetic vesicles. (**a**) Nanovesicles with isolated proteins are produced by top-down or bottom-up approaches by isolating a specific protein from a biological source and putting it into the lipid bilayer of a nanovesicle to confer a specific function to the delivery system. (**b**) Biological nanovesicles, such as small extracellular vesicles and outer membrane vesicles, can be used directly as drug or gene delivery systems; macrovesicles, such as cell and bacteria ghosts, can be extracted and downsized by extrusion or sonication for use in this application also. (**c**) Hybrid nanovesicles are obtained from the fusion of a biological vesicle and a synthetic liposome by physical or chemical methods in order to increase production yield.

**Figure 4:**
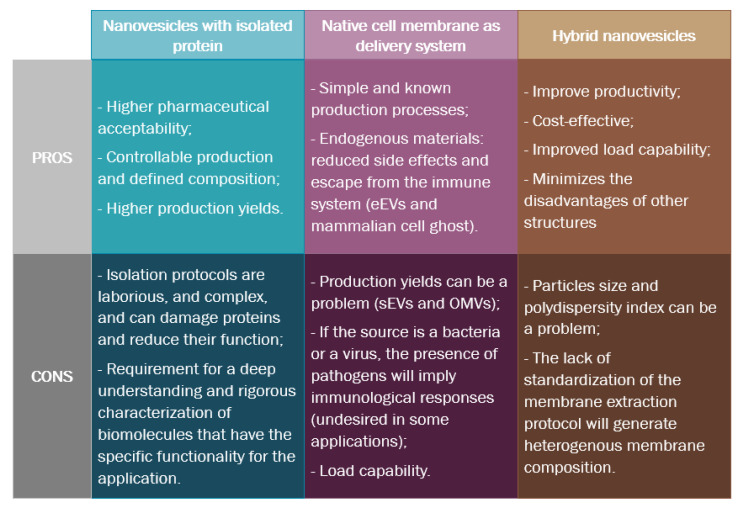
Advantages and disadvantages of each design strategy for biomimetic nanovesicles.

Different steps must be overcome to design biomimetic nanovesicles (BNV) (Figure 3), and each has its advantages and disadvantages (Figure 4).

It is important to specify the application, define the natural membrane source and their extraction protocol, and decide on the protocol for the BNV synthesis. BNV can be obtained using conventional protocols to obtain synthetic liposomes and by adding specific proteins that confer special properties (Figure 3a). In this case, the specific protein needs to be isolated and purified. Native cell membranes can be used as BNV by extracting and downsizing protocols. Encapsulation protocols can be used according to the specific application. As an alternative, the camouflage strategy allows the “encapsulation” of a specific nanoparticle using the cell membrane components (Figure 3b). The third alternative promotes hybridization between natural components from different membrane sources and synthetic liposomes (Figure 3c). An overview of the main features addressed in the literature regarding the biomimetic nanovesicle designs described can be found in Table 1. These three strategies are described in the following sections.

### 3.1. Liposomes Containing Isolated Protein from Cell Membranes 

It is well-known that plasma proteins easily opsonize conventional liposomes, and that they lack stability and confer low circulating time in intravenous injection [58]. Then a known phenomenon is the corona protein formation around the liposomes, which forms plasma proteins around a nanoparticle in a physiological environment. This protein “crown” is formed by the high charge of the surface of a nanoparticle. This protein formation can vary in healthy and sick individuals and from one disease to another [59]. This interaction at the interface of a nanoparticle with the biological environment was one of the motivations for specialists to develop nanovesicles like natural cells, that is, with the modification in their composition by adding cell membrane proteins in phosphocholine-based phospholipids [60]. Thus, the corona protein formed on the surface of nanoparticles could be one of the key points modulating nanoparticles’ biological identities for delivery to specific locations [59]. 

The corona protein that forms around the nanoparticle to carry it in the bloodstream can mediate the biodistribution of this nanocarrier and the drug delivery. In a comparative study, it was seen that liposomes with leukocyte membrane proteins in their composition absorb fewer corona proteins. This promotes their circulation and neutralizes their uptake by blood vessels by in vivo immune cells, unlike conventional protein-free liposomes. According to the authors, this surface modification may function as leukocyte receptors favoring the iteration, with immunoglobin G (IGg) antibody and protein receptors not being able to bind their receptors to those of macrophages, which is what happens to liposomes without modifications [61].

Therefore, researchers have developed liposomes capable specifically targeting properties, such as membrane proteins of specific cells, for treating diseases. The method of manufacturing these nanostructures consisted of extraction of the desired protein and the addition of this protein, which would provide a specific objective to the liposome membrane during its manufacture through a micro-scale channel of equipment called Nano Assembler [60,62]. This methodology will be explored further below.

It is also possible to associate this method with adding polyethylene glycol (PEG) chains to the surface of liposomes modified with proteins, improving the colloidal stability. Zinger et al. (2021) developed nanovesicles modified with cell membrane proteins as tools to improve the specificity of liposomes for treating neurological diseases in different lipid formulations: one consisting of Dipalmitoylphosphatidylcholine (DPPC), 1,2-dioleoyl-sn-glycero-3 -phosphocholine (DOPC), and cholesterol (4:3:3 molar ratio) and the other of 1,2-dipalmitoyl-3-dimethylammonium-propane (DAP), DSPE-PEG2000, and cholesterol (4.2:1:4.8 molar ratio). Using the cell membrane protein of human pluripotent stem cells (hPSCs) requires the use of specific protocols for the extraction, purification, and quantification of the proteins. When comparing the presence of polyethylene glycol (PEG), the authors observed differences in surface loads between the two formulations (the first with PEG reduced the zeta potential and the second without PEG kept the neutral surface, increasing the zeta potential). According to the authors, this effect caused an increase in in vitro selective targeting, with the hypothesis being that long PEG chains hinder cell uptake by protein iterations, preventing the adsorption of proteins on its surface and promoting greater tissue penetration [62].

The addition of specific proteins in the liposomes’ composition confers advantages to the final BNV, facilitating the nanoaggregate synthesis protocol. Conversely, there is a need to identify the protein precisely, and it requires a deep knowledge of the cell membrane proteins and their function for the appropriate selection. In addition, there is also a need to develop new processes that can recover and purify these membrane proteins in a specific way, promoting a high process complexity.

### 3.2. Native Cell Membranes as a Delivery System

Different sources of membranes can be used as delivery systems, from extracellular vesicles to downsized membrane compounds (ghost cells). In this case, the membrane source is the only material to obtain the BNVs. 

The use of extracellular vesicles as a delivery system first requires proper extraction and purification. Then, the drug or gene material has to be encapsulated using conventional techniques, as described in the following sections. Curcumin, a bioactive pigment of turmeric, can be loaded into exosomes to treat diseases like cancer, oxidative stress, and brain disorders [63,64]. OMVs loaded with doxorubicin showed an antitumor effect in vivo associated with an immune response, serving as a chemoimmunotherapy agent [63].

The cell membrane can be used as the delivery system as an alternative to extracellular vesicles. In this case, proper purification and downsizing are important process steps. The advantage of using the cell membrane itself is the increase in its biospecificity, since transmembrane proteins act as beacons when delivering drugs to a target site. In this case, the probability of drug delivery is much higher. In addition, studies that used nanoghosts and mesenchymal cell membrane nanovesicles were able to cross different biological barriers and deliver the drug or active ingredient to some regions of the cell (membrane, cytoplasm, and nucleus) [48,49].

As an alternative, conventional nanoparticles can be covered by cell membrane components. A cell without cytoplasm has been studied to encapsulate nanoparticles or active ingredients to form a core–shell structure. The terminology to define this structure has not yet been fully established by the authors and can be called membrane-coated nanoparticles [56], core–shell [64], or even membrane camouflage [65]. However, all these biomimetic nanostructures have the characteristic of going unnoticed through biological barriers and delivering drugs to treat diseases. Using cell membranes as a coating for nanocarriers was extensively explored as a promising platform in the review by Liu et al. (2019) [66].

**Table 1:** Important features regarding the biomimetic nanovesicle designs.

Nanostructure	Membrane Source	Targeting	Important Features	Refs.
Nanosized native cell membrane	Mesenchymal stem cells (MSCs) membrane (hypotonic lysis)	Cancer	Requires large number of cells (compare to the hybrid nanovesicle approach)	[48,49,67]
			Expensive method relative to membrane source	
			The delivery system for cancer treatment can circumvent resistance to therapy	
			Ability to cross biological barriers	
Native membrane cloaked nanoparticle	Platelet membrane (freeze–thaw process)	Atherosclerosis	Good system evasion (increased adhesion of platelets with endothelial cells)	[50,68,69,70]
			Less complex extraction protocol (anucleated cells)	
			Requires large number of cells (compare to the hybrid nanovesicle approach)	
			Target for antithrombosis drug delivery	
			Good immune evasion of platelet membranes	
	Monocyte cell membrane (hypotonic lysis)	Cancer	Ability to retain biological targeting properties of the cell of origin	[67]
			Higher cellular uptake in cancer cells	
			Good targeting for cancer cells	
			Ability to make intercellular homologous bonds with membrane proteins	
			Good targeting for cancer cells	
			Cancer cell membrane coating improved; sustained release of chemotherapeutic and decreased cellular uptake of macrophages	
Biomimetic vesicles with isolated proteins (proteolipid)	Leukocyte plasma membrane proteins	Inflammation target	Referred to as leukosomes by the authors	[60,71]
			Can bypass macrophages (increased circulation)	
			Difficulty obtaining (needs more blood to obtain)	
			Lack of targeting for tumor targeting	
			Compared to conventional liposomes, leukosomes preferentially recognized the inflamed endothelium	
	Genetically engineered hPSC differentiating in neurons plasma membrane proteins	Neurodegeneration	Referred to as neurosomes by the authors	[62]
			Ability to mediate protein-protein interactions with target cells	
			Bottom-up microfluidics-based synthesis method to make nanovesicles	
			The ability to circulate in the blood or cross the blood–brain barrier (BBB) has not been evaluated	
Hybrid nanovesicle Membrane	Platelet membrane (Freeze–thaw process)	Atherosclerosis	Requires less amount of cells compared to coating (low cost)	[50,70]
			Good targeting of inflammation cells, immune evasion of platelet membranes, biocompatibility and biodegradability when fusing with liposomes, prolonged circulation and ability to encapsulate and release components,	
	Mesenchymal stem cells (MSCs) membrane (Freeze–thaw)	Inflammation tropism to ischemic cerebrum	MSCs can communicate with immune cells like phagocytes and microglia; ability to cross the blood–brain barrier (BBB); prolonged circulation and ability to encapsulate and release components	[55,72]
			Ability to reduce uptake by the mononuclear phagocytic system	
	Exosomes (MSC-derived)	Cancer	Good result of polyethylene glycol (PEG)-mediated fusion of cell-derived EVs with liposomes bringing biocompatible compositions	[73]
			Can add biogenic functionalities to the liposome for enhanced cellular absorption	
			Promotes increased circulation and improved targeting of permeability and retention	
	Exosomes (macrophage-derived)	Cancer	Good drug delivery to cancer cells under acidic conditions	[74]
			Ability to target sick/inflammatory cells due to surface proteins acquired by EV	
			Nanovesicle surface proteins acquired by EV play a significant role in intercellular adhesion	
	Genetically engineered exosomes (CD47-expressed)	Cancer	Able to penetrate the target tumor and release loads under hypothermic conditions of HIPEC after intravenous administration	[75]
			May surpass the efficiency of delivering chemotherapeutic drugs to large tumor nodules	
Two hybrid nanovesicle membranes	Leukocyte–tumor cell plasma membrane (hypotonic lysis)	Cancer	Increased affinity and uptake with tumor cells	[54]
			Prolonged blood circulation	
			Multiple biofunction for drug delivery	
			High loading capacity for poorly water-soluble anticancer drugs.	
Two native membrane-cloaked nanoparticles	Erythrocyte (hypotonic lysis) -platelet (freeze–thaw) plasma membrane	Blood circulation	Ability to load features from both source cells	[65,76,77]
			Circulation for long periods of time	
			Presents immunomodulatory markers, such as CD47, with density similar to original red blood cells	

The process to obtain membrane-coated nanoparticles occurs primarily with the extraction of the cell membrane. This can be done via several methods, including freeze–thaw, hypotonic treatment, and ultrasound. Then, there is the encapsulation of another non-specific nanoparticle with a drug or active ingredient inside, serving as a coating, and only the cell membrane functioning as a carrier [48,49].

The nanoparticle-cell communication can be improved by using the biospecificity of a membrane cell protein in nanoparticles. The CD47 cell membrane protein mediates in circulation, so macrophages do not clear the nanoparticle. Wang et al. (2019) propose a drug delivery system for atherosclerosis by a biomimetic structure with the red cell membrane (red blood cells). In this study, the nanostructure comprises the camouflage of a nanoparticle of the immune system’s poly(lactic-co-glycolic) acid (PLGA). This strategy was based on a proposition that the membrane protein of the red blood cell CD47 regulates phagocytosis by macrophages through receptors [64]. In addition, functionalization of membranes with an adaptation of specific ligands increases the target direction of these nanocarriers [78].

Many in vitro and in vivo results have been investigated using membrane cells as nanovesicle or camouflage systems for nanoparticles. However, one of the main disadvantages of using only cell membrane components is that it increases the necessary number of cells and, consequently, the total cost. An alternative to decrease the number of cells and standardize the process is hybridizing the natural membranes with liposomes. Studies show that, using only cell membranes, it takes approximately 3 million more cells to camouflage a PLGA nanoparticle than fusing liposomes [50,65].

### 3.3. Liposome and Native Membrane Hybrid Nanostructure

Fusing or hybridizing animal cell membranes (extracellular vesicles or membrane cell compounds) with synthetic liposomes, unlike surface modification with specific components, is a process that does not require many purification steps after membrane extraction. In addition, it is cheaper when compared to the use of native membranes as it requires fewer cells and can be mixed with commercial lipids. The cell membrane and synthetic liposome are similar in their structural components since they both have a phospholipid bilayer vesicle, and many authors have used this advantage to hybridize these structures. In addition, the hybrid liposome becomes more biospecific for medical applications with the cell’s natural proteins and lipids. The approach of merging different membranes, whether natural cell membranes or liposomes, is also considered camouflage by some authors [79]. Applying similar characteristics of the target cell structure and going unnoticed in the delivery of active components is also an objective of this approach.

When we compare final hybrid liposomes, as BNVs obtained, with purified lipids and only one membrane protein or a membrane pool, this last one increases the biomimetic characteristic of the nanovesicle. In this case, the protein poll confers more than one specific characteristic to the BNV. This approach does not require steps of extraction and characterization of proteins to be incorporated, still maintaining the stability of the liposome and the ability to encapsulate drugs [50,55]. A lower cost can be obtained using native membrane components as a carrier, since, in a study with the use of platelet cell membrane, it was demonstrated to be necessary for around 3 × 109 platelets for coating and 2 × 109 platelets to hybridize with liposomes [50,65]. The disadvantage of this method comes in terms of the standardization of the membrane extraction protocol so it can become scalable, since so far, this has only been developed on a laboratory scale. Also, there may be a lack of standardization of the membrane as it is natural, so the process is naturally not homogeneous— in terms of size, properties, or its general heterogeneity.

There is also the possibility of a fusion or hybridization of two different cell membrane types of cells with liposomes, increasing the biospecificity of the final BNV. One example is a study that fused the plasma membrane of tumor cells with the plasma membrane of leukocytes, conferring two functions to nanoparticles and improving cancer treatment. The nanovesicle improved the blood circulation by dribbling the immune system due to leukocytes and acquiring a more significant target and absorption capacity to the target tumor. The process consists of extracting the plasma membranes, forming a dispersion with both, and then fusing these membranes with the phosphatidylcholine membrane through the extrusion process. The result is a modified liposome with layers of phosphatidylcholine lipids and membrane components from two different cells [54]. Using two types of membranes it is possible to promote the advantage of the nanocarrier, obtaining at least two specific characteristics for its targeting. One is the ability to reach specific sites expected by that nanovesicle due to the specific biomarkers of the source cell. The other is the increase in circulation time because it goes unnoticed by the immune system [79,80].

## 4. Biomimetic Nanovesicle Production Methods

In this section, the main concepts in nanotechnology fabrication will be discussed. Then, the methods applied for biomimetic nanovesicles synthesis will be presented. 

### 4.1. Strategy—Top-Down/Bottom-UpTop-Down and Bottom-Up

Bottom-up and top-down concepts encompass all areas of nanotechnology manufacture and synthesis. Likewise, biomimetic nanovesicles can be produced by these two approaches, and naturally, a third one: the combination of top-down and bottom-up. 

#### 4.1.1. Top-Down Method

Top-down methods starts at the macro or micro scale and apply techniques to reduce the particle size to the nanoscale [46,81]. By analogy, it is like an artist sculpting a block of stone to create the desired image. Biomimetic nanovesicles produced by top-down approach are cell-derived nanovesicles, mammalian cell and bacterial ghosts, and some hybrid nanovesicles, and some hybrid nanovesicles.

The main advantage of this method is that it brings to the final product the composition complexity of the source, much like a membrane of a cell-derived nanovesicle has the same composition as the mother cell. Also, these methods are well-known and easily scalable for industrial applications. The downside is related to the final size and size distribution, and the lack of control of these parameters that can lead to the necessity to add a new step in the production process. Additionally, it is important to evaluate encapsulation and drug retention when these methods are employed [46,81]. Some examples of unit operations commonly applied to produce biomimetic nanovesicles like cell-derived nanovesicles, bacterial and cell ghosts, and hybrid nanovesicles are extrusion, sonication, and high-shear mixing.

#### 4.1.2. Bottom-Up Method

By contrast, the bottom-up method assembles nanostructures from the atomic or molecular scale, similarly to joining many pieces to compound a puzzle. This approach takes advantage of supramolecular interactions that self-assemble nanostructures at specific conditions (i.e., hydrophilic–hydrophilic, and hydrophobic–hydrophobic interactions, hydrogen bonding, van der Waals, electrostatic, among others) [46,81].

Bottom-up procedures synthesize nanostructures with a high degree of control over size and size distribution. Nevertheless, obtaining raw materials for these processes requires complex purification protocols that can damage proteins and affect their functionality. Furthermore, by using these methods it is hard to reproduce the whole complexity of lipid and protein composition of biological membranes. Thus, bottom-up approaches require a profound understanding of each biomolecule incorporated to achieve the appropriate application and function [46,81].

Solvent injection and microfluidics are examples of bottom-up methods already used to produce drug delivery systems. 

As can be seen above pros and cons of each technique are complementary. Thus, combining top-down and bottom-up methods naturally emerges as a strategy to overcome the limitations of each approach.

### 4.2. Physical Methods

#### 4.2.1. Freeze and Thaw

The freeze and thaw (or freeze–thaw) is a classic method and can be used to fuse membrane bilayers, produce ghosts, and load vesicles [55,82,83,84]. This method consists of quickly freezing the sample in liquid nitrogen at −80 °C and thawing at room temperature, and repeating this cycle usually between 5 to 15 times. The process variables in this method are the number of cycles, the mass concentrations or ratios, and membrane composition.

There are two hypotheses to explain the phenomena: (1) the ice crystal mechanically breaks the bilayer membrane or (2) gradient solubility formed during the transient stage of the phase change [85,86]. Moreover, the effect on the bilayer membrane can be transient or permanent. 

This technique’s advantages are simple, rapid, low cost, and does not require technical skill or specialized instruments. The downside of this method is that it leads to multilamellar structures, induces nanovesicle aggregation, increases the polydispersity index, and changes the size distribution [82,85]. The freeze–thaw method cannot be applied in a continuous flow process, which is another disadvantage of this technique.

#### 4.2.2. Sonication

Sonication is a technique that applies acoustic waves at the frequency of ultrasound (20 kHz–1 MHz) to destabilize the bilayer membrane, increase permeability, and even disrupt it. The disruption happens because cavitation phenomena occur when acoustic waves interact with water molecules. Bubble formation, growth, and collapse produce local instability in bilayer membranes [82,87,88].

The sonication process variables are the number of cycles, time with sonication on and off, and frequency [82]. Typically, sonication is applied pulsed (also named cycles) to avoid the increase in temperature and its harmful effects on biological compounds.

This technique has already produced liposomes, nanoghosts, hybrid nanovesicles by membrane fusion, and load vesicles [48,74]. The main advantage of this technique is that it can be used in a continuous flow process [89]. 

#### 4.2.3. Extrusion

In the extrusion procedure, the fluid that contains the particles is forced through a nanopores membrane. If the particles are larger than nanopore size the lipid bilayer will disrupt and rearrange in a closer membrane size [90]. It is usually passed around 20 to 10 times on membranes of pore size between 800 nm, 400 nm, 200 nm, and 100 nm, and can be either cellulose acetate membranes or polycarbonate membranes [50,54]. This method is one of the most widely used to produce nanoparticles or to suit ‘particles’ size and morphology.

Extrusion can be used to turn multilamellar vesicles (MLVs) into monodisperse unilamellar vesicles [89,90]. Also, the method can be applied to fuse different types of nanoparticles; for instance, it can fuse liposomes with ghosts delivered from platelet membranes [50].

#### 4.2.4. Incubation

Sometimes the simple incubation between two nanoparticles species is sufficient to fuse them. In this case, there is an artifact behind the phenomenon, like electrostatic attraction or some functionalized molecule into the membrane in one of the vesicles. Incubation also has promoted drug and gene encapsulation. Agitation is an important artifice to increasing extracellular vesicle and OMV production [91,92]. Usually, the incubation time varies from minutes to hours, and the interval more frequently seen in studies is 4 h [63,93,94,95].

### 4.3. Chemical Methods

#### 4.3.1. Polyethylene Glycol

Polyethylene glycol (PEG) has been known and used for a long time for bilayer fusion, for example for producing hybridomas. PEG is a polymer with a high affinity with water (hydrophilic) and when in the medium it competes with the lipids on bilayers, causing dehydration. This change in hydration leads to structural modification and then fusion [96].

Hybrid nanovesicles were produced by the fusion of synthetic liposomes and extracellular vesicles from different cell sources. The fusion process was mediated by polyethylene glycol (PEG) and added at a final concentration of 5–30% (*w*/*v*) [73]. 

#### 4.3.2. DOPE—1,2-Dioleoyl-sn-Glycero-3-Phosphoethanolamine

Dioleoylphosphatidylethanolamine (DOPE) is known as a “helper lipid” and incorporated at 10% to 20% molar in the liposome formulations helps the nanostructure to fuse with another bilayer. DOPE spontaneously assumes the inverted hexagonal (H_II_) phase when present in the lipid bilayer. This configuration has been proposed as an important mechanism for membrane fusion [97,98]. Unlike others phosphatidylethanolamines, DOPE has both acyl chains unsaturated which causes reduced mobility of the phospholipid tails, as well as decreases the effective head group area [99,100]. In other words, it is related to its critical packing parameter [101] and will influence the membrane fluidity by tending to form the inverted hexagonal (H_II_) phase.

Many studies report increased transfection efficiency when DOPE is used in cationic liposome formulation. Some hypotheses have been proposed to explain the DOPE helper effect in transfection: the inverted hexagonal (H_II_) phase destabilizes the endosomal membrane, the neutral charge can weaken the binding between cationic lipids and DNA, and the pH responsive structural transition of DOPE that can coincide with the endosomal maturing [102].

#### 4.3.3. Calcium–Mediate Fusion

Divalent cations are an old candidate to promote fusion between vesicles and have been investigated since the early seventies. Calcium has the best results, especially when the membrane contains phosphatidylserine, cardiolipin, and phosphatidylcholine. There is evidence that Ca+2 promotes the transition to the inverted hexagonal (HII) phase in certain phospholipids. As is well known, the bilayer–hexagonal transition helps the membrane fusion process. During the phenomena also occurs leakage of inner content, the mixture between the contents of the vesicles fused, the interchange of lipids and proteins from the bilayer membrane, and aggregation of vesicles [103,104,105,106].

Calcium was recently used to fuse liposomes with cell-delivered plasma membrane vesicles (CDV) from HEK293T cells. The mother cells employed to produce the vesicles were genetically engineered to overexpress a membrane protein, and the vesicles were produced by incubation with cytochalasin B. It is important to note that the liposomes have DOPE in their composition, which is a fusogenic agent, too [106].

### 4.4. Continuous Flow Methods

#### Microfluidics

Liposomes have been explored in the field of microfluidics by many study groups [107]. This methodology explores a bottom-up approach to achieve greater control of the desired size and polydispersity without requiring a later size reduction step (i.e., extrusion, sonication, and others) [107]. Microfluidics takes advantage of a laminar flow in the microchannel (low Reynolds number) together with the phenomenon of mass transfer (diffusion) with control of lipid concentration and total flow rates through the hydrodynamic focusing method [108]. To achieve this process configuration, a microdevice is developed with a microchannel of 100 μm deep and 140 μm wide, approximately. Two aqueous phases in the lateral input channels are inserted to form streams flowing in parallel, and lipids are dispersed in ethanol at the central entrance. These parallel streams will promote the gradual ethanol/water mixing and, consequently, lipid self-aggregation and liposome formation. In addition, the width of the cross-section of the flow achieved by hydrodynamic focusing will depend on the control of the Flow Rate Ratio (FRR) in the input currents and the total volumetric flow rate (Qt) in the microchannel output current [109]. These characteristics make microfluidics a unique tool for pharmaceutical and biotechnological applications related to nanotechnology.

However, because it is a laminar flow governed by diffusion, some applications that require an increase in mass transfer can be impaired, such as obstructions in the canals by the formation of unexpected microstructures due to the low mixing process of the components [110]. To overcome this limitation, a passive mixture can be applied through the phenomenon of chaotic advection [107]. For this, some barriers are projected in the microchannel with standardized geometries modulating the fluid to control the mixture and flow with chaotic characteristics, but still in a laminar regime [107]. There are several geometry configurations for the micromixer design depending on the application and the final characteristics of the nanovesicle. One study increased productivity by up to 70 times. It used a microdevice based on chaotic advection in herringbone configuration compared to hydrodynamic, focusing on liposome formation with average diameters of approximately 200 nm and with low polydispersion [69]. Chaotic advection can also be explored to achieve a higher area/volume ratio, an increase in mass production, and increased flow rates in the microchannel, achieving an increase in the yield of nanovesicle formation, thus overcoming diffusion limitations [111].

Molinaro et al. (2018) and Zinger et al. (2021) reproduce through a bottom-up process; the microfluidic synthesis of liposome nanovesicles are modified with a cell membrane protein [60,62]. The process consists of a microfluidic device (NanoAssembler^®^) forming a hydrodynamic focus with two inputs: an aqueous phase with dispersed membrane proteins and the other with lipids and cholesterol dispersed in ethanol. The microdevice has a microchannel with a y-shaped configuration using a standardized configuration barrier called herringbone mixers. This configuration increases hydrodynamic focus with the phenomenon of chaotic advection. This phenomenon forces the fluid to circumvent standardized obstacles with stretching and unfolding volumes providing an increase in diffusion between fluids and functioning as a fine adjustment micromixer of the final physical-chemical characteristics of the nanovesicles. These conditions are ideal for forming nanovesicles in a high-yield bottom-up approach in gentle flow conditions, reducing thermal exchange interference and shear forces [48,69].

The authors can obtain a biomimetic nanovesicle with a specific biological function in the exit channel, conferring a modified liposome [60]. This strategy was designed to standardize the control of final properties in scalable lots in manufacturing these biomimetic nanovesicles. Some parameters facilitate the achievement of this objective: (i) the control of the flow rate ratio (TRR) in the microchannel of the two phases, i.e., between the aqueous and the solvent phase; (ii) the control of the total flow rate (TFR) in the microdevice; (iii) control of the temperature to be operated and (iv) control of lipid concentration and proteins to be obtained in the nanovesicle at the microchannel output by self-aggregation of both lipids and cell membrane proteins [50,69,109,110].

It is noted that there is still a vast field to be explored in developing of biomimetic nanovesicles in bottom-up approaches. In this context, the microfluidic synthesis of biomimetic nanocarriers production presents future opportunities in different approaches.

## 5. Applications for Drug and Gene Delivery, and Vaccine

The design and strategy selected to create biomimetic nanovesicles define the drug delivery system’s functionality and application. The membrane source, the culture conditions, and the method of synthesis and loading set the nanoparticle behavior and biospecificity. As seen below, different biomimetic nanovesicles have been used in important fields of pharmaceutics to treat challenging diseases.

### 5.1. Cancer Therapy

In an increasingly polluted world with people’s lives characterized by unhealthy lifestyles, the occurrence of cancer has dramatically increased and is one of the leading causes of death. Among the available treatments, chemotherapy and immunotherapy are effective approaches for cancer treatment. These therapies gain even more importance when conventional treatments such as surgery are not possible, or in more severe cases of the disease [112,113,114].

Chemotherapy drugs are very cytotoxic and kill both cancer and healthy cells. The nonspecific action of chemotherapeutic agents leads to physical pain and harmful effects on human health [112,114]. In this sense, a drug delivery system is essential for successful cancer treatment. Several studies have shown biomimetic nanoparticles’ potentiality for cancer treatment.

A thermosensitive hybrid between exosomes and liposomes was applied as a chemotherapy carrier for metastatic peritoneal cancer treatment. The exosomes were derived from fibroblast cells genetically engineered to overexpress CD47 membrane protein. Exosomes and thermosensitive liposomes were generated by fusion using the freeze and thaw method. The nanostructure was loaded with the granulocyte-macrophage colony-stimulating factor (GM-CSF) and docetaxel, effectively inhibiting tumor development in vivo [75].

Ghosts from 4T1 cells, breast cancer cells, were employed as a carrier of paclitaxel (PTX) to treat metastatic cancer in mouse models. It produced the ghost of 4T1 cells by sonication and 400 nm extrusion polycarbonate membrane. After, it loaded the chemotherapeutic PTX by 200 nm polycarbonate membranes. This biomimetic vesicle targeted the primary tumors and the metastatic nodules in mouse models and has improved the circulation time compared with other carriers [115].

The immune system is a powerful mechanism that works to maintain the organism health, seeking and destroying both external and internal threats. There are pieces of evidence that the immune system fights against cancer cells. However, cancer sometimes perform a series of complex maneuvers to escape the immune system. Tumors create a microenvironment, use cytokines and growth factor secretion, and cellular signaling to evade and suppress the immune system’s response. Thus, immunotherapy emerges to teach the immune system to recognize and fight tumors. Some strategies to achieve this goal include vaccination, oncolytic viruses, or immune checkpoint inhibitors [112,113,116,117,118]. For immunotherapy, have been proposed different kinds of biomimetic vesicles.

PROVENGE^®^ (sipuleucel-T) (Dendreon Corporation) is a biomimetic vesicles FDA-approved drug to treat metastatic castrate-resistant prostate cancer. The technology consists of a cancer vaccine based on autologous peripheral blood mononuclear cells associated with prostatic acid phosphatase (prostate antigen). However, the efficacy of increasing patient survival time was questionable [113,119].

Biomimetic nanovesicles synthesized through the fusion between Salmonella outer membrane vesicles (OMV) and melanoma cells have shown promising results for antitumor vaccination. The B16F10 melanoma cells have been used to produce ghosts by hypotonic disruption and differential centrifugation. The OMVs from *Salmonella* were isolated from the culture medium by centrifugation. Then, the fusion process was done by sonication and extrusion of the OMVs and cell ghost mixture, and the fusion performance was measured by Förster resonance energy transfer (FRET). The core was loaded by extrusion with indocyanine green (ICG) and poly(lactic-co-glycolic acid) polymeric nanoparticles. Intradermally immunized mice can stimulate the immune system to have a specific response against cancer [120].

A virus-like particle (VLP) carrying a photoactivatable drug was used to treat the tumor and exhibit residual antitumor immunity in the TC-1 syngeneic murine tumor model. Human papillomavirus 16 (HPV16) was employed to produce de VLP, and the drug-loaded was IRDye 700DX (IR700; LI-COR Biosciences). About half of the mice treated showed long-term antitumor immunity and rejected subsequent tumors [121].

One of the major challenges that immunotherapy faces off against is the great heterogeneity of cancer, which remains valid for biomimetic nanovesicles.

### 5.2. Gene Delivery

Viruses, OMVs, bacteria, extracellular vesicles, and cells endogenous carry nucleic acid into their core. This innate characteristic can help to build a gene delivery system with enhanced specific targeting, long circulations time, and nucleic acid protection.

Hybrid extracellular vesicles and liposomes were used to carry CRISPR–Cas9 expression vectors and transfect mesenchymal stem cells (MSCs), on a condition that small extracellular vesicles or liposomes could not transfer the genes. The hybrid was synthesized by simple incubation between small extracellular vesicles delivered from HEK293FT cells and Lipofectamine 2000. The plasmid was first loaded to Lipofectamine 2000, then fused with small extracellular vesicles. Note that the authors use exosomes to describe what actually are small extracellular vesicles [91].

Extracellular vesicles (EV) from genetically engineered HEK-293T cells were used as gene delivery carriers in in vivo studies [122]. Another work also employs extracellular vesicles to deliver siRNA in mice in which the EV mother’s cells were fibroblast L929 [123]. Also, a report uses bacterial ghosts to deliver si/shRNA in vivo [124].

### 5.3. Brain Delivery

Biological barriers are one of the most efficient apparatuses for organs and tissues’ protection against pathogens and diseases. Some examples can be mentioned: blood–brain barrier (BBB), stromal barrier, placental barrier, and blood–air barrier, among others. Nonetheless, when an organ or tissue protected by these barriers gets sick, it is challenging to deliver drugs, even for nanometric vesicles [125,126,127]. No doubt, crossing the blood–brain barrier is one of the major challenges faced by modern drug delivery systems and pharmacology.

In recent studies, biomimetic nanovesicles are being explored as candidates to cross the blood–brain barrier. Engineered or not, small extracellular vesicles are the most common vesicles employed to this aim, probably because of their endogenous origin.

Small extracellular vesicles derived from macrophages were used as a carrier to cross the blood–brain barrier in mice models. The nanovesicles were loaded with a protein cargo, the brain-derived neurotrophic factor, by simple incubation. This was possible since cargo and carrier have electrostatic attraction. Even though a small fraction of the injected dose (0.093 ± 0.02 %D/g brain -0.538 ± 0.315 %ID/g brain), the study showed that small extracellular vesicles are candidates for brain drug delivery systems [92].

A liposome containing an isolated protein, apolipoprotein E (ApoE), was used as a carrier to deliver α-mangostin in vivo. The nanoparticle was synthesized by thin-film hydration method followed by sonication, and the drug was loaded during this process. Fluorescent imaging analysis showed a higher signal in mice brain when compared to the control [128].

Virus-inspired nanocarriers can cross the blood–brain barrier, especially as a gene delivery system. Viruses like Herpes simplex virus type 1 (HSV-1), lentivirus, adenovirus, and adeno-associated virus were used as vectors for drug and gene delivery [129].

### 5.4. Vaccines

Vaccines-related issues have gained massive attention in the last two years due to the COVID-19 pandemic. Indeed, major pandemics like the Spanish flu, cholera, and the Black Death has already challenged humanity. Over time, science evolved, and new technologies emerged, like rapid diagnostic testing and contact tracing [130]. Among all advances, rapid development and production of vaccines plays a crucial role in pandemic responses. Biomimetics nanovesicles can be designed to serve as a vaccine.

A vaccine proactively stimulates the human immune system, teaching it how to defend itself from the pathogen without causing the disease. Among the desirable characteristics is the presence of an antigen from the pathogen capable of inducing an immune response without causing side effects. Another important parameter that influences vaccine performance is particle size [131,132].

VLP and virosomes are candidates for vaccine development since they carry the antigen from the parent virus. Examples of VLPs already approved by the FDA are Gardasil^®^ (Merck), and Cervarix^®^ (GlaxoSmithKline), both human papillomavirus bivalent (Types 16 and 18) vaccines [46]. The virosomes approach also has FDA-approved vaccines, like Invivac^®^ (Solvay-Influenza) and Inflexal V^®^ (Crucell) for the influenza viruses and Epaxal^®^ (Crucell) against the hepatitis A virus [17,25,133,134,135]. A series of vaccine virus-inspired candidates are in development against infections of SARS-CoV-2, hepatitis B virus (HBV), Varicella–Zoster virus, Ebola virus, and Zika virus, among others [17,22,25].

OMVs are well-known vaccine candidates, as shown by several studies over the last 20 years. They have the appropriate size for this application, and since their biogenesis is derived from the membrane of Gram-negative bacteria, they carry various antigens from the pathogens. In 2015, the FDA approved an OMV Neisseria meningitidis serogroup B vaccine, the BEXSERO^®^ (GlaxoSmithKline) [136]. Various inspired OMV vaccine candidates are being studied against *S. pneumoniae* (serotype 14), *Mycobacterium tuberculosis*, *V. cholera*, *H. pylori*, *Salmonella typhimurium*, and *Shigella flexneri* (*S. flexneri*), among other viruses [25,28,33,136]. The most common challenge reported in using OMV as a vaccine is reactogenicity, especially that caused by lipopolysaccharides (LPS). Engineering and genetic engineering of these biomimetic nanovesicles can be a way to address these challenges.

A new heterologous vaccine candidate against meningitis and Zika virus was developed by Martins, el at. 2018. The group created a hybrid nanoparticle by the fusion of *Neisseria meningitidis* OMVs and Zika virus (ZIKV) derived from infected C6/36 cells. The fusion was achieved by mechanical force (agitation), which is easily scalable and decreases the cost of vaccine production. This engineered biomimetic nanovesicle was tested on mice, and ELISA and inflammatory chemokines protocols evaluated their immune response. Also, the serum from mice immunized with the hybrid nanoparticles significantly decreased the virus RNA copy number in the results of the in vitro soroneutralization test [22]. This is an example of how engineering biomimetic vesicles can address public health challenges.

A novel vaccine type has emerged in the last years as an efficient tool against infections: DNA vaccines. These vaccines are composed of genes that, when administrated lead to the expression of a foreign antigen, activating the immune system. A drug delivery system must carry a successful DNA vaccine. Thus, the genes are protected against nuclease degradation, the uptake by the cell is facilitated, and the circulation lifetime is prolonged [137,138]. Initial studies involving biomimetic structures as carriers reveal promising results. VLP carrying a plasmid encoding human immunodeficiency virus env (HIV env) induced an immune response in mice [139]. Other studies have also shown that bacterial ghosts and animal erythrocyte ghosts carried genes at in vitro tests [140,141]. 

## 6. Conclusions and Future Perspectives

As far as science evolves, a profound understanding emerges of diseases like cancer, central nervous system diseases, heart disease, and infectious diseases, among others. Furthermore, the rate of these diseases is expected to increase in an increasingly polluted world in combination with people’s unhealthy lifestyles. Thus, a drug and gene delivery system with precise, specific targeting is essential to ensure that lower doses of the drug reach the site at the right dose for the proper therapeutic effect.

There is a clear upward trend in using biomimetic nanovesicles as drug and gene delivery systems and as vaccines. Although this field of pharmaceutics has been explored for years, it is only in the last two decades that research has grown exponentially, and the first drugs have been approved. Improving biospecific characteristics, site-specific targeting, and loading capability to prolong circulation time by using this natural source of vesicles is a smart strategy and requires a proper understanding of the application and source properties.

Thus, the native cell membrane or only some biomolecules from different biological sources, like exosomes, small extracellular vesicles, OMV, and ghost cells, can be used depending on the application. Although there are some biomimetic nanovesicles already approved, much work is ahead to identify the best combination of membranes or isolated proteins, and to develop and scale up the production processes. We envision, for example, these biomimetic nanovesicles, that can serve as a vector for cell lineage edition using CRISPR/Cas9 and miRNAs, to manipulate cell lines to drive development or prevent uncontrolled proliferation when necessary. In reproduction, removing undesired genotypes or disease-associated genotypes can be one mechanism to continue increasing the precision of genetic gain.

Biomimetic nanovesicles have been produced at most on laboratory benches, but industrial processes must be developed when the technology takes off and becomes financially viable and commercially Continuous flow processes are more attractive to industry; in this sense, there is an unexplored field to develop. 

In this sense, the development of biomimetic nanovesicles requires a multidisciplinary team since biologists understand the functional biomolecules of the biological sources, nanotechnology and biotechnology specialized engineers are needed to synthesize biomimetic nanovesicles and develop production processes, and medics are required for evaluating the effects on the human body. Thus, combining the advantages of synthetic systems, like controllability and mass production, with the endogenous characteristics and functions of biological vesicles is a robust strategy to address the main challenges that pharmaceutics will face in the drug and gene delivery field systems and vaccines. New technologies must be developed to allow the design of the magic bullet in the future, following the vision of Paul Ehrlich for over 100 years.

## Abbreviations

ApoE	Apolipoprotein E
BBB	Blood–brain barrier
BG	Bacterial ghost
BNV	Biomimetic Nanovesicles
CDV	Cell-delivered plasma membrane vesicles
COVID-19	Coronavirus Disease 2019
DAP	1,2-dipalmitoyl-3-dimethylammonium-propane
DNA	Deoxyribonucleic acid
DOP	1,2-dioleoyl-sn-glycero-3-phospho-L-serine (DOPS)
DOPC	1,2-dioleoyl-sn-glycero-3 -phosphocholine
DOPE	Dioleoylphosphatidylethanolamine
Dox	Doxorubicin
DOXIL	Doxorubicin (liposomal)
DPPC	Dipalmitoylphosphatidylcholine
DSPC	1,2-Distearoyl-sn-glycero-3-phosphocholine
DSPE-PEG2000	1,2-distearoyl-sn-glycero-3-phosphoethanolamine-N-[amino(polyethylene glycol)-2000]
EPC	Egg Phosphatidylcholine (1,2-dioleoyl-sn-glycero-3-ethylphosphocholine)
ESEs	Early sorting endosomes
EV	Extracellular vesicles
FDA	Food and Drug Administration
FRET	Förster resonance energy transfer
HIV	Human immunodeficiency virus
hPSCs	Human pluripotent stem cells
HPV16	Human papillomavirus 16
HSV-1	Herpes simplex virus type 1
ICG	Indocyanine green
IGg	Immunoglobin G
ILVs	Intraluminal vesicles
LPS	Lipopolysaccharide
LSE	Late sorting endosomes
MAP	Model amphiphilic peptide
MSCs	Mesenchymal stem cells membrane
MVB	Multivesicular bodies
NP	Nanoparticle
OMV	Outer Membrane Vesicles
PC S100	Soya Phosphatidylcholine
PE	Phosphatidyl ethanolamine
PEG	Polyethylene glycol
PG	Phosphatidyl glycerol
PLGA	Poly(DL-lactide-co-glycolide)
PNP	PLGA nanoparticles
POPC	1-palmitoyl-2-oleoyl-sn-glycero-3-phosphocholine
PPG	Peptidoglycan
PTX	Paclitaxel
RNA	Ribonucleic acid
sEVs	Small extracellular vesicles
VLP	Virus-like particles
